# Supplementary material for: Thermal Stability Enhancement of L-Asparaginase from Corynebacterium glutamicum Based on a Semi-Rational Design and Its Effect on Acrylamide Mitigation Capacity in Biscuits
Source: Foods. 2023 Dec 3;12(23):4364. doi: 10.3390/foods12234364 (PMC10706719; doi:10.3390/foods12234364)
Supplement: Supplementary file 1 [file foods-12-04364-s001.zip › foods-2664698-supplementary.pdf]

Supplementary Materials for

Thermal Stability Enhancement of L-Asparaginase from *Corynebacterium glutamicum* Based on a Semi-Rational Design and Its Effect on Acrylamide Mitigation Capacity in Biscuits

Huibing Chi †, Qingwei Jiang †, Yiqian Feng, Guizheng Zhang, Yilian Wang, Ping Zhu, Zhaoxin Lu and Fengxia Lu *

College of Food Science and Technology, Nanjing Agricultural University, Nanjing 210095, China; t2023091@njau.edu.cn (H.C.); 2021121002@stu.njau.edu.cn (Q.J.); 2022808095@stu.njau.edu.cn (Y.F.); 2022108054@stu.njau.edu.cn (G.Z.); 2021808098@stu.njau.edu.cn (Y.W.); pingzhu@njau.edu.cn (P.Z.); fmb@njau.edu.cn (Z.L.)

* Correspondence: lufengxia@njau.edu.cn; Tel.: +86-25-84395963

† These authors contributed equally to this work.

ORCID

Zhaoxin Lu: 0000-0001-9288-7419

Fengxia Lu: 0000-0003-0934-7847

Ping Zhu: 0000-0002-4393-7608

Table S1. Oligonucleotide primers used in site-point saturation mutagenesis

Numbers	Sequences (5′→3′)
I67-F	CCTTCGAA**NNK**CATGAAATCAATCGCCTGGATA
I67-R	TTCATG**MNN**TTCGAAGGCGATCTGGGCGCCAT
I70-F	TCCATGAA**NNK**AATCGCCTGGATAGCAGCAGC
I70-R	GCGATT**MNN**TTCATGGATTTCGAAGGCGATCT
N71-F	ATGAAATC**NNK**CGCCTGGATAGCAGCAGCATG
N71-R	CAGGCG**MNN**GATTTCATGGATTTCGAAGGCGA
N59-F	TTC**NNK**GGCGCCCAGATCGCCTTCGAAATCCA
N59-R	ATCTGGGCGCC**MNN**GAAGCGCGGGGCGATC
V100-F	GGATGTT**NNK**GGCGTTGTTGTTACCCATGGCA
V100-R	CAACGCC**MNN**AACATCCGGATCTTCCAGAACTT
N146-F	CCG**NNK**AATCTGTTCGAAGCCTGCCTGATCGC
N146-R	TCGAACAGATT**MNN**CGGGCCATCGGCTTCCGG
C152-F	TTCGAAGCC**NNK**CTGATCGCCAGCGATCCGAG
C152-R	ATCAG**MNN**GGCTTCGAACAGATTATTCGGGCC
E187-F	ATACCAGCGAT**NNK**CTGGCCTTCGCCACCAAT
E187-R	CAG**MNN**ATCGCTGGTATGCCATTTAACGCAGC
K259-F	GGGC**NNK**GCCCTGGATGCCGGCATCCCGGTTGTT
K259-R	ATCCAGGGC**MNN**GCCCAGGGCATCGCCCATGC
F302-F	TCGGGAGCCGCTAT**NNK**CGCGCCGGCCAAGCCCGG

Numbers	Sequences (5′→3′)
F302-R	**MNN**ATAGCGGCTCCCGACGGCGCCTTTGGCGG
T120-F	CGCCGTTGAT**NNK**TTCCTGGATGATCCGCGCC
T120-R	GGAA**MNN**ATCAACGGCGATGGCGCTTTCTTCC
S213-F	CGATGTT**NNK**GTTGAAATTATCCCGGCATATCC
S213-R	TTTCAAC**MNN**AACATCGGCCAGTTTGGCAACC
T224-F	GGTGCA**NNK**GGTGCCATGGTGGAGGCCGCCAT
T224-R	ATGGCACC**MNN**TGCACCCGGATATGCCGGGAT
H171-F	TTTCGGC**NNK**GCCGTTATCCCGGCCCGCGGCT
H171-R	TAACGGC**MNN**GCCGAAAACGATCAGGGCGCCG
K294-F	AACACTCGCCGCC**NNK**GGCGCCGTCGGGAGCCGC
K294-R	C**MNN**GGCGGCGAGTGTTGCTCCGCCGCCTGCA
Y325-F	CCATCCGGTTACCCTG**NNK**CTCGAGG
Y325-R	CCTCGAG**MNN**CAGGGTAACCGGATGG
K135-F	CCCAG**NNK**CCGTTCGATCATCCGGAAGCCGAT
K135-R	ATCGAACGG**MNN**CTGGGCCCCGGTGAAGATAA
M227-F	ACGGGTGCC**NNK**GTGGAGGCCGCCATAGCGGC
M227-R	TCCAC**MNN**GGCACCCGTTGCACCCGGATATGC
H40-F	CAATGGC**NNK**CTTCTGCCGACCGTCAGCGGGG
H40-R	GCAGAAG**MNN**GCCATTGGCATCGCTGGTGCAG
I313-F	TCCTGCTAGCC**NNK**GCCATCGCGACCGG

Numbers	Sequences (5′→3′)
I313-R	CCGGTCGCGATGGC**MNN**GGCTAGCAGGA
S251-F	ATGTTGGC**NNK**CGCATGGGCGATGCCCTGG
S251-R	CATGCG**MNN**GCCAACATTGCCGCTGCCCATGG
M269-F	GGTTGTT**NNK**AGCACCCGAGTTCCGCGCGGAG
M269-R	GGGTGCT**MNN**AACAACCGGGATGCCGGCATCCA
L42-F	CACCTT**NNK**CCGACCGTCAGCGGGGCTGATCT
L42-R	ACGGTCGG**MNN**AAGGTGGCCATTGGCATCGCT
C179-F	GC**NNK**GTTAAATGGCATACCAGCGATGAACTG
C179-R	ATGCCATTTAAC**MNN**GCCGCGGGCCGGGATAAC
K208-F	C**NNK**CTGGCCGATGTTAGCGTTGAAATTATCC
K208-R	TAACATCGGCCAG**MNN**GGCAACCGGCAGGGCATC
Q62-F	**NNK**ATCGCCTTCGAAATCCATGAAATCAATCG
Q62-R	ATTTCGAAGGCGAT**MNN**GGCGCCATTGAAGCGCGG
F65-F	CC**NNK**GAAATCCATGAAATCAATCGCCTGGAT
F65-R	TTCATGGATTTC**MNN**GGCGATCTGGGCGCCATT

*The mutation sites were underlined and highlight.

Table S2. Orthogonal experimental factors and levels

Level	Factors		
	A: Enzyme dosages (IU/kg)	B: Enzyme reaction times (min)	C: Enzyme reaction temperatures (℃)
1	100	30	30
2	300	40	40
3	500	50	50

Table S3. Combination of different processing conditions for three variable and three level orthogonal experiments

Numbers	A: Enzyme dosages (IU/kg)	B: Enzyme reaction times (min)	C: Enzyme reaction temperatures (℃)
1	100	30	30
2	100	40	50
3	100	50	40
4	300	30	40
5	300	50	50
6	300	40	30
7	500	40	40
8	500	50	30
9	500	30	50

Table S4. Potential stabilizing mutation sites, recommended mutant amino acids, and threshold values of CgASNase predicted by Consensus Finder

Numbers	Sites	Amino acids in CgASNase	Amino acids in consensus sequence	Frequency of amino acids in the consensus sequence
1	302	F	L	89%
2	120	T	L	80%
3	213	S	R	73%
4	171	H	G	71%
5	224	T	D	71%
6	294	K	A	68%
7	325	Y	F	66%
8	135	K	R	65%
9	67	I	V	59%
10	70	I	L	51%
11	65	F	V	51%
12	227	M	L	48%
13	40	H	A	47%
14	313	I	A	44%
15	269	M	V	42%
16	42	L	V	40%

Numbers	Sites	Amino acids in CgASNase	Amino acids in consensus sequence	Frequency of amino acids in the consensus sequence
17	251	S	P	43%
18	259	K	R	37%
19	187	E	L	35%
20	100	V	D	35%
21	152	C	I	33%
22	71	N	M	30%
23	59	N	L	30%
24	146	N	R	31%
25	179	C	V	28%
26	208	K	P	28%
27	62	Q	G	26%

Table S5. Analysis of results of orthogonal experiments

Numbers	A: Enzyme dosages	B: Enzyme reaction times	C: Enzyme reaction temperatures	D: Empty column	Acrylamide reduction (%)
1	1	1	1	1	80.51±1.93[d]
2	1	2	3	2	85.14±2.84[c]
3	1	3	2	3	89.97±1.89[b]
4	2	1	2	2	92.35±2.03[a]
5	2	3	3	1	90.02±0.58[b]
6	2	2	1	3	92.01±1.18[a]
7	3	2	2	1	75.42±2.81[e]
8	3	3	1	2	76.64±2.96[e]
9	3	1	3	3	63.35±3.92[f]
K1	85.307	78.737	83.067	80.317	
K2	91.473	82.537	84.247	84.710	
K3	70.137	85.543	79.503	81.790	
R	31.336	6.806	4.744	4.393	RA>RB>RC>RD

Table S6. Analysis of variance (ANOVA) for results of orthogonal experiments

Source of variation	Deviation sum of squares	Degree of freedom	F ratio	F critical value	Significance
A	721.639	2	24.056	19.0	*
B	69.811	2	2.327	19.0	
C	36.589	2	1.220	19.0	
Errors	30	2			

Note: * indicates a significant difference between samples (p<0.05).

Table S7. Effect of different enzymatic reaction times on the inhibition of acrylamide in biscuits

Number	A: Enzyme dosages (IU/kg)	B: Enzyme treatment times (min)	C: Enzyme treatment temperatures (℃)	Acrylamide reduction（%）
A2B1C2	300	30	40	92.35±2.03[a]
A2B2C2	300	40	40	92.87±1.81[a]
A2B3C2	300	50	40	93.02±1.86[a]
